# Case report: Multisystem inflammatory syndrome in children associated with COVID-19, macrophage activation syndrome, and incomplete Kawasaki disease

**DOI:** 10.3389/fped.2023.1167828

**Published:** 2023-04-17

**Authors:** Jesús Javier Martínez-García, Jesús Ramon López-Acosta, Daniela Arce-Cabrera, Nidia León-Sicairos, Ana Cristina Hernández-Parra, Hector Melesio Cuen-Diaz, Ricardo Zatarain-Lopez, Adrian Canizalez-Roman

**Affiliations:** ^1^ Pediatric Intensive Care Unit, Pediatric Hospital of Sinaloa, Culiacan, Mexico; ^2^ School of Medicine, Autonomous University of Sinaloa, Culiacan, Mexico; ^3^ Hematology and Oncology Unit, Pediatric Hospital of Sinaloa, Culiacan, Mexico; ^4^ Research Department, Pediatric Hospital of Sinaloa, Culiacan, Mexico; ^5^ FCA, Autonomous University of Sinaloa, Culiacan, Mexico; ^6^ Research Department, The Women’s Hospital, Secretariat of Health, Culiacan, Mexico

**Keywords:** multisystemic inflammatory syndrome in children (MIS-C), SARS-CoV-2, COVID-19, macrophage activation syndrome (MAS), Kawasaki disease (KD)

## Abstract

**Background:**

Multisystem inflammatory syndrome in children (MIS-C), is a severe complication of coronavirus disease 2019 (COVID-19), characterized by persistent fever, systemic inflammatory response, and organ failure. MIS-C with a history of COVID-19 may share clinical features with other well-defined syndromes such as macrophage activation syndrome, Kawasaki disease, hemophagocytic syndrome and toxic shock syndrome.

**Case 1:**

An 11-year-old male with a history of hypothyroidism and precocious puberty with positive antibody test for COVID-19 was admitted for fever, poor general condition, severe respiratory distress, refractory shock, and multiple organ failure. His laboratory examination showed elevated inflammatory parameters, and bone marrow aspirate showed hemophagocytosis.

**Case 2:**

A 13-year-old male with a history of attention deficit hyperactivity disorder and cognitive delay presented clinical manifestations of Kawasaki disease, fever, conjunctival congestion, exanthema, and hyperemia in oral mucosa, tongue, and genitals, with refractory shock and multiple organ failure. Reverse transcriptase polymerase chain reaction (RT-PCR) and antibodies for COVID-19 were negative, inflammation parameters were elevated, and bone marrow aspirate showed hemophagocytosis. Patients required intensive care with invasive mechanical ventilation, vasopressor support, intravenous gamma globulin, systemic corticosteroids, low molecular weight heparin, antibiotics, and monoclonal antibodies and, patient 2 required renal replacement therapy.

**Conclusions:**

Multisystemic inflammatory syndrome in children can have atypical manifestations, and identifying them early is very important for the timely treatment and prognosis of patients.

## Introduction

During the second half of April 2020, a syndrome probably related to severe acute respiratory syndrome coronavirus 2 (SARS-CoV-2) infection in pediatric patients was described for the first time in European countries (United Kingdom and Italy) and in the United States (New York) as multisystem inflammatory syndrome in children (MIS-C) (1–5). MIS-C is a rare and severe complication of coronavirus disease 2019 (COVID-19). The World Health Organization (WHO) and the Center for Disease Control and Prevention (CDC) issued the case definition criteria for MIS-C. They require the presence of fever, elevated inflammatory markers, at least two signs of multisystem involvement, and evidence of infection or exposure to SARS-CoV-2 in the 4 weeks prior to symptom onset (6–9). However, the Royal College of Paediatrics and Child Health criteria for the diagnosis of MIS-C8, although similar but not identical to those described by WHO and CDC, admit the possibility of negative reverse transcriptase polymerase chain reaction (RT-PCR) testing for SARS-CoV-2 and make no reference to antibodies to COVID-19. This new syndrome presents with symptoms similar to Kawasaki disease (KD), toxic shock syndrome (TCS), secondary hemophagocytic lymphohistiocytosis (HLH), and macrophage activation syndrome (MAS) (6–8, 10).

In this study, we describe the clinical features, laboratory data, and treatment of two patients with MIS-C and macrophage activation syndrome in the first case and Kawasaki disease in the second case.

## Case presentation

### Case 1

**Figure 1:**
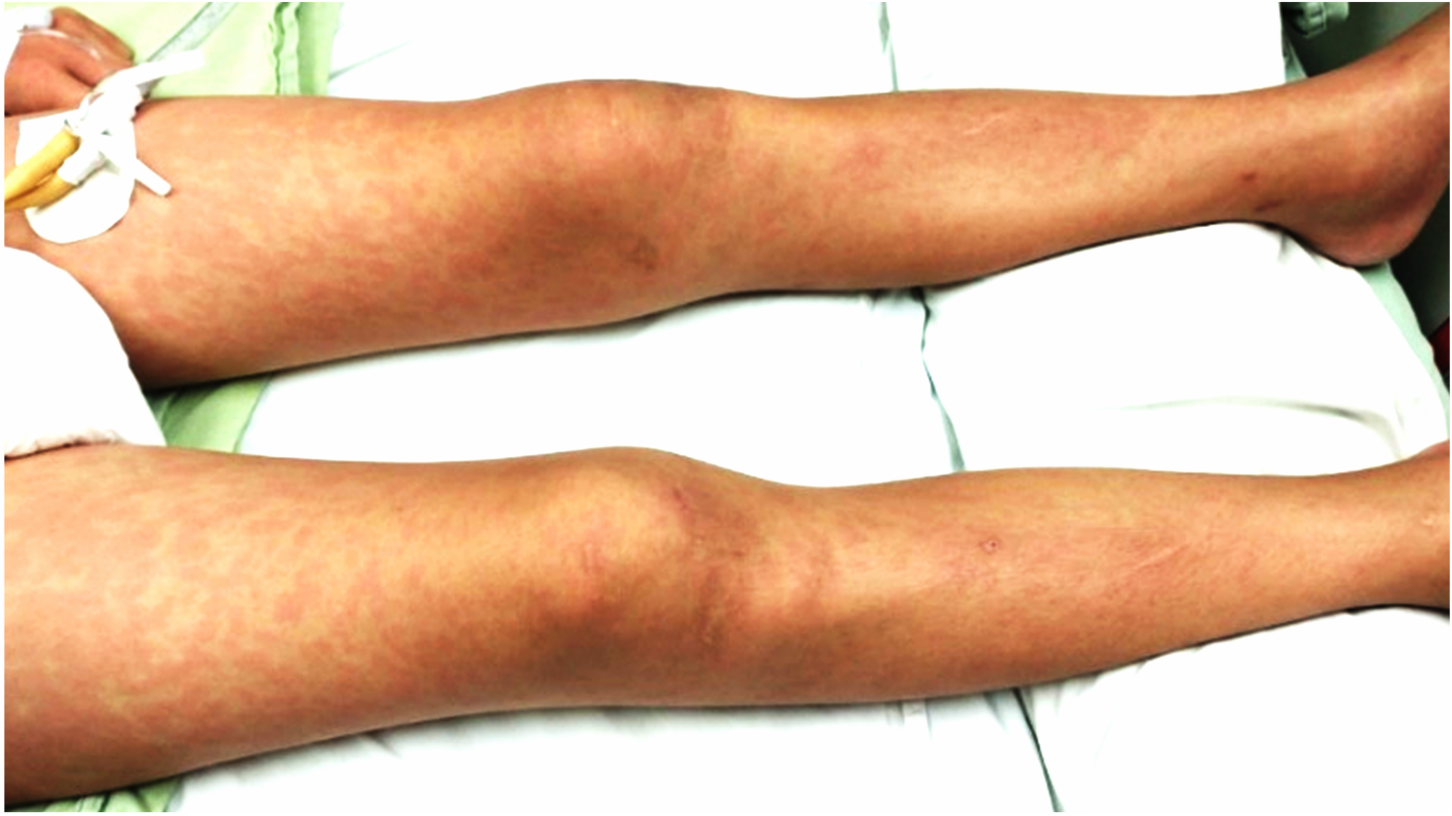
Maculopapular rash on the lower extremities.

Our first case was an 11-year-old boy with a history of hypothyroidism and precocious puberty diagnosed at 3 and 10 years of age, respectively, under treatment with levothyroxine and leuprolide. One month prior to his hospitalization, he presented contact with his father with clinical manifestations and positive real-time reverse transcriptase polymerase chain (PCR-RT) for COVID-19. The clinical picture began with upper respiratory tract infection, abdominal pain, vomiting, no diarrhea, fever >39°C for 5 days, rash on trunk and extremities (Figure 1), jaundice, and chest pain.

He was admitted to the pediatric emergency department with clinical symptoms of decompensated hypovolemic shock, treated with 0.9% saline loads, mechanical ventilation, and inotropic support with dobutamine. Initial laboratory findings with systemic inflammatory response, negative nasopharyngeal RT-PCR for COVID-19, and positive antibodies for COVID-19 interpreted a diagnosis of MIS-C. He was admitted to the pediatric intensive care unit (PICU) in a poor general condition with cardiogenic shock and significant hemodynamic compromise. Color Doppler echocardiogram showed pericardial effusion, mitral insufficiency, left ventricular ejection fraction (LVEF) of 32%, and no coronary dilatation (left coronary: 3.8, *Z* score +5; anterior descending: 3.1, *Z* score +1. circumflex: 2.3, *Z* score +0.5). A bone marrow aspirate was performed and showed macrophages phagocytizing platelets and lymphocytes (Supplementary Figure S1).

He was treated with intravenous immunoglobulin (IVIG) at 2 g/kg/dose, methylprednisolone at 30 mg/kg/dose for 3 days, a second dose of IVIG at 1 g/kg/dose, baricitinib 4 mg/dose every 24 h, and enoxaparin at 1 mg/kg/day. He received inotropic and vasopressor support milrinone, levosimendan, adrenaline, and noradrenaline. He presented with supraventricular tachycardia that improved with synchronized cardioversion, adenosine, and continuous infusion of esmolol. Evolution was favorable, with normalization of clinical and laboratory alterations. He remained on mechanical ventilation for 5 days, cardiovascular support for 8 days, and hospitalization in the PICU for 10 days. The patient was started on an oral polymer feed 48 h after admission to the PICU.

### Case 2

**Figure 2:**
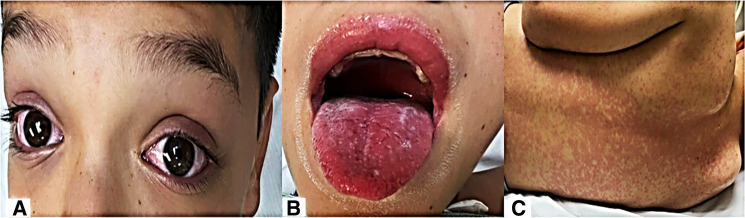
(**A**) Bilateral nonsuppurative conjunctival injection; (**B**) hyperemic oral mucosa and tongue; and (**C**) erythematous maculopapular rash.

Our second case was a 13-year-old male with a history of attention deficit hyperactivity disorder and cognitive delay diagnosed at age 5 years, receiving treatment with methylphenidate. He had a history of contact with his father with COVID-19 1 month before admission. Clinical picture of 4 days of evolution, with cough, rhinorrhea, odynophagia, headache, and fever up to 39°C of difficult control; conjunctivitis (Figure 1); abdominal pain, diarrhea and vomiting; hyporexia; and erythematous maculopapular exanthema on trunk and extremities, was obtained. Physical examination showed conjunctival injection; hyperemia of lips, tongue, and pharynx (Figures 2A–C); neck adenomegaly, genital hyperemia; and inguinal adenomegaly.

The patient was admitted to the pediatric emergency department with clinical symptoms of decompensated distributive shock; initial treatment was 0.9% saline and dobutamine. Laboratory results showed a systemic inflammatory response; negative serological and RT-PCR (nasal swab sample) results were obtained for SARS-CoV-2; even with these results, a diagnosis for MIS-C was established according to the Royal College of Paediatrics and Child Health criteria. He presented with poor evolution due to refractory shock to fluids and amines, the airway was secured, and he was admitted to the PICU and started receiving oral polymer-supplemented nutrition 48 h later.

Color Doppler ultrasound showed pulmonary pressure of 38 mmHg, pericardial effusion, LVEF 57%, no coronary dilatation (left coronary: 3.1, *Z* score −0.4; anterior descending: 2.8, *Z* score +0.16; circumflex: 3.5, *Z* score +1.67). A bone marrow aspirate was performed and hemophagocytosis was observed (Supplementary Figure S2). He was treated with IVIG at 2 g/kg/dose and methylprednisolone at 30 mg/kg/dose for 3 days. A second dose of IVIG at 1 g/kg/dose, tocilizumab at 9.5 mg/kg/dose, and enoxaparin at 1 mg/kg/day was administered. He received treatment with sildenafil and received inotropic and vasopressor support with milrinone, levosimendan, adrenaline, noradrenaline, and vasopressin. He received renal replacement therapy for 4 days. The patient evolved favorably; he remained on mechanical ventilation for 9 days, cardiovascular support for 10 days, and hospitalization in the PICU for 11 days. The clinical manifestations of the two patients are compared with the clinical manifestations described for MIS-C, MAS, and KD in Table 1, and the laboratory findings of the two cases are described in Table 2.

**Table 1:** Comparison of the clinical features of MIS-C, MAS, and KD with the two cases described.

Clinical manifestations	MIS-C	MAS	KD	Case 1	Case 2
Age (years old)	∼10–11	Any age	<5	11	13
Prevailing gender	Male	Any gender	Male	Male	Male
Etiology or complication of the disease	SARS-CoV-2	Cancer, SLE, JIA, KD, infections, immunodeficiency	Autoimmune diseases	SARS-CoV-2	Family history with COVID-19
Fever ≥39°C	≥3 days	≥5 days	≥5 days	≥3 days	≥3 days
Non-purulent conjunctivitis	+	−	++	−	++
Rash	Maculopapular	Purpuric	Polymorphic	Maculopapular	Maculopapular and erythema
Arthritis	+	−	+	+	−
Throat pain	+	−	+	+	+
Oral mucosa inflammation	+	−	++	−	+
Headache/irritability	+	++	+	+	+
Lymphadenopathy	+	+	++	+	++
Abdominal pain	++	−	−	+	+
Diarrhea	++	−	−	−	+
Vomiting	++	−	−	+	+
Hand and feet edema	+	−	+	−	+
Peeling feet	+	−	+	−	+
Shock	++	++	−	+	++
Respiratory distress	+	+	−/+	++	+
Acute kidney injury	+	+	−/+	+	++
Heart failure	++	+	++	++	+

MIS-C, multisystem inflammatory syndrome in children; MAS, macrophage activation syndrome; SLE, systemic lupus erythematosus; JIA, juvenile idiopathic arthritis; KD, Kawasaki disease; SARS-CoV-2, severe acute respiratory syndrome coronavirus 2; COVID-19, coronavirus 19 disease.

**Table 2:** Comparison of laboratory studies between the two cases of MIS-C.

Laboratory parameters	Case 1	Case 2
Hemoglobin, g/dl	10.5	12.2
Hematocrit, %	31.4	37
Leukocytes, 10^3^/μl	9.91	5.2
Total neutrophils	9,117	4,330
Lymphocytes	299	611
Neutrophil/lymphocyte ratio	30.4	7.0
Platelets, 10^3^/μl	109	191
Prothrombin time	11.9	16.7
Partial thromboplastin time	30.6	34.5
Ferritin, ng/ml	2,422	1,830
Fibrinogen, mg/dl	510	480
D-dimer, ng/ml	14,600	5,000
Troponin, ng/ml	0.07	0.03
Sodium, mEq/L	139	143
Potassium, mEq/L	5.0	4.3
GOT, U/L	95	297
GPT, U/L	112	127
Total bilirubin, mg/dl	4.05	2.8
Triglycerides, mg/dl	569	313
Urea, mg/dl	74	108
Creatinine, mg/dl	0.7	1.5
C-reactive protein, mg/dl	17.4	22.9
Procalcitonin, ng/dl	15.58	41.26
Brain natriuretic peptide, pg/ml	25,000	16,830
Interleukin-6, pg/ml	35	5,000
Lactate dehydrogenase, U/L	876	445
Dengue (IgG, IgM, Ag NS1)	−	−
Leptospira	ND	−
Rickettsia	ND	−
Blood culture	−	−
PCR-RT COVID-19	−	−
IgG, IgM, COVID-19	+	−
Echocardiogram	Pericardial effusion Mitral insufficiency LVEF 32%	Pericardial effusion Pulmonary hypertension LVEF 54%
Chest x-ray/CT	Pneumonia with pleural effusion	Pneumonia with pleural effusion
Bone marrow aspirate	Macrophage aspiration syndrome	Hemophagocytic Sx

GOT, glutamic oxaloacetic transaminase; GPT, glutamic pyruvic transaminase; PCR-RT, real-time reverse transcriptase polymerase chain; IgG, immunoglobulin G; IgM, immunoglobulin M; LVEF, left ventricular ejection fraction; ND, not determined; MIS-C, multisystem inflammatory syndrome in children; COVID-19, coronavirus 19 disease.

## Discussion

In this report, we describe two cases of MIS-C: The first patient had a history of COVID-19 4 weeks before admission to the PICU and positive antibodies (immunoglobulins, IgG and IgM) for COVID-19, with clinical features and bone marrow aspirate results suggestive of MAS. The second patient had only with a family history of COVID-19, but was with RT-PCR and antibodies (IgG, IgM) negative for COVID-19, with clinical features compatible with KD and bone marrow aspirate result with hemophagocytosis.

Multisystem inflammatory syndrome presents with some clinical and laboratory features similar to KD, TCS, secondary hemophagocytic lymphohistiocytosis (SHLH), and MAS (6–8). Most publications include only patients with confirmed SARS-CoV-2 infection, although a few case reports include children with hyperinflammatory shock from other causes (9, 10). To date, there are no well-defined criteria for classifying the severity of MIS-C, although there are reviews that describe the factors that should be taken into account to classify MIS-C as severe: admission to PICU, organ failure (renal, hepatic, respiratory, cardiac), fluid-refractory hypotension, noninvasive or invasive ventilation, and support with inotropic drugs (10).

To date, there are no well-defined criteria for classifying the severity of MIS-C, although there are reviews that describe the factors that should be taken into account to classify MIS-C as severe: admission to PICU, organ failure (renal, hepatic, respiratory, cardiac), fluid-refractory hypotension, noninvasive or invasive ventilation, and support with inotropic drugs.

These patients presented with hemophagocytosis, which is defined as the uptake of blood cells, including red blood cells, white blood cells, or platelets, by phagocytic cells. Hemophagocytosis by macrophages has been widely associated with the development of MAS in patients with juvenile idiopathic arthritis and other rheumatologic diseases (11, 12). During the acute phase of MAS, it is associated with elevated levels of proinflammatory cytokines. This cytokine storm triggers a cascade of inflammatory pathways that, if left unchecked or untreated, lead to tissue damage and death. The leading hypothesis suggests that macrophages and monocytes produce significant amounts of cytokines, mainly tumor necrosis factor (TNF) and several interleukins (IL-6, IL-1β, and IL-18), which triggers a cascade of inflammatory pathways, thus creating a cytokine storm (11–14).

The first patient met the criteria for MAS described above: IL-6 levels were 35.7 pg/ml, which could have been higher, especially since they were taken 4 days after initiation of steroid and immunoglobulin therapy, and was supported by bone marrow aspirate where hemophagocytosis of platelets and lymphocytes was observed.

Since the first described cases of COVID-19, the number of cases with Kawasaki-like disease in patients with COVID-19 has increased considerably (15). MIS-C has clinical manifestations that resemble incomplete or atypical KD. In typical KD, generally 85% of cases have been observed in infants and children under 5 years of age, although it can occur in older children (16). The main American Heart Association criteria for KD are fever equal to or greater than 5 days in 100%, nonsuppurative bulbar conjunctivitis in 95%, thickened hyperemic lips with fissures with hyperemic tongue (strawberry) in 90%, polymorphous exanthema in 76%, and cervical lymphadenopathy greater than 1. The main cardiac manifestations are arteritis and coronary artery aneurysm, although endocarditis, pericardial effusion, and myocarditis may be observed (16, 17).

The term incomplete KD is applied when the primary criterion of fever equal to or greater than 5 days plus other criteria supporting the diagnosis is met, having excluded other diseases that may mimic this disease without meeting all the criteria for complete KD. Incomplete KD is also considered when the criteria are met with the exception of persistent fever (17, 18).

Another term used to classify KD is atypical KD, which is used when there are one or more atypical features of KD, such as pulmonary involvement (pneumonia), arthritis, nephritis or renal failure, myositis, uveitis, retinal vasculitis, and central nervous system involvement (facial paralysis and meningoencephalitis) (17, 18).

According to these definitions, the second patient had clinical manifestations compatible with atypical KD, fulfilling all the American Heart Association clinical criteria for KD. The patient had elevated acute phase reactants, with a very significant increase in IL-6, and also had acute kidney injury and was receiving renal replacement therapy. In this patient, RT-PCR and antibodies for COVID-19 were negative, with a family history of COVID-19, although the Royal College of Paediatrics and Child Health criteria for the diagnosis of MIS-C admit the possibility of having MIS-C with a negative RT-PCR for COVID-19.

Patients with MIS-C receive empirical treatment, based on Kawasaki disease treatment protocols. The recommended treatment for MIS-C is IVIG at high doses of 2 mg/kg/day in divided doses. Glucocorticoids are part of the initial treatment, in patients with a severe clinical presentation; our patient, with significant cardiovascular involvement and evidence of MAS, was treated with methylprednisolone pulses at 30 mg/kg/day for 3 days, followed by 2 mg/kg/day in divided doses (19, 20).

In patients with left ventricular dysfunction documented by an LVEF <35%, anticoagulation with enoxaparin is warranted (19, 21). The first patient was admitted to the PICU in cardiogenic shock with an LVEF of 32% and a considerable increase in inflammatory/cardiac markers (C-reactive protein, IL-6, ferritin, D-dimer, troponin, creatine phosphokinase, and natriuretic peptide). Severe cases of refractory MIS-C are usually treated with a second dose of IVIG or anakinra, a recombinant interleukin-1 (IL-1) receptor antagonist, which blocks IL-1α and IL-1β, key players in the “cytokine storm” and hyperinflammatory response of MIS-C (19, 21).

Other treatments used in MIS-C that have not shown satisfactory results in MIS-C morbidity and mortality include infliximab, which is a human chimeric monoclonal antibody that blocks tumor necrosis factor-alpha (TNF-α), is frequently used to treat refractory cases of Kawasaki disease (1, 21). The first patient was treated with baricitinib, an inhibitor of Janus kinases (JAK-1 and JAK-2). JAK is a tyrosine kinase that mediates cytokine activation signals. Janus kinase inhibitors can effectively suppress and inhibit the cytokine storm and have been used to treat various autoimmune and hematological diseases.

Baricitinib inhibits hyperinflammation in COVID-19 pneumonia, including inhibition of proinflammatory mediator release and virus endocytosis (19, 20). In the second patient, he received treatment with tocilizumab, a humanized monoclonal antibody against the IL-6 receptor, which prevents IL-6 from binding to its receptor to exert IL-6-promoted immunosuppression (1, 4).

## Conclusions

MIS-C is a potentially life-threatening inflammatory disease; late recognition of symptoms, if not adequately treated, can lead to multiorgan failure and death. Our patients had MIS-C, a severe complication of COVID-19, supported by WHO and CDC criteria; the first patient had clinical and laboratory manifestations and a bone marrow aspirate that confirmed the diagnosis of MAS, and the second patient had clinical manifestations of atypical Kawasaki disease.

## Data Availability

The datasets generated and/or analyzed during the current study are available from the corresponding author upon reasonable request.
