# Supplementary figures and images for: Case report: Multisystem inflammatory syndrome in children associated with COVID-19, macrophage activation syndrome, and incomplete Kawasaki disease
Source: Front Pediatr. 2023 Apr 17;11:1167828. doi: 10.3389/fped.2023.1167828 (PMC10149839; doi:10.3389/fped.2023.1167828)

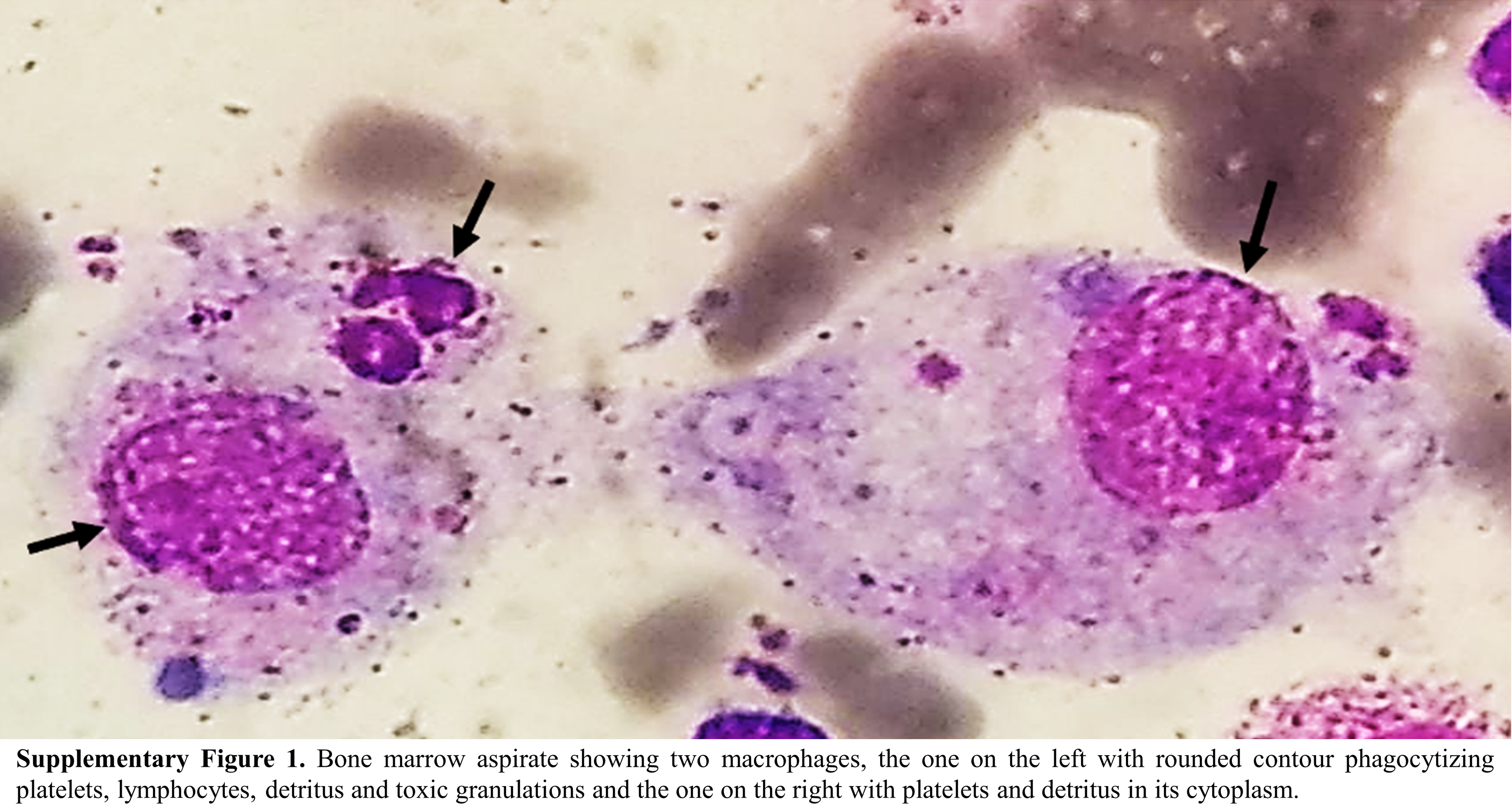

Supplement: Supplementary file 1 [file Image1.tif]

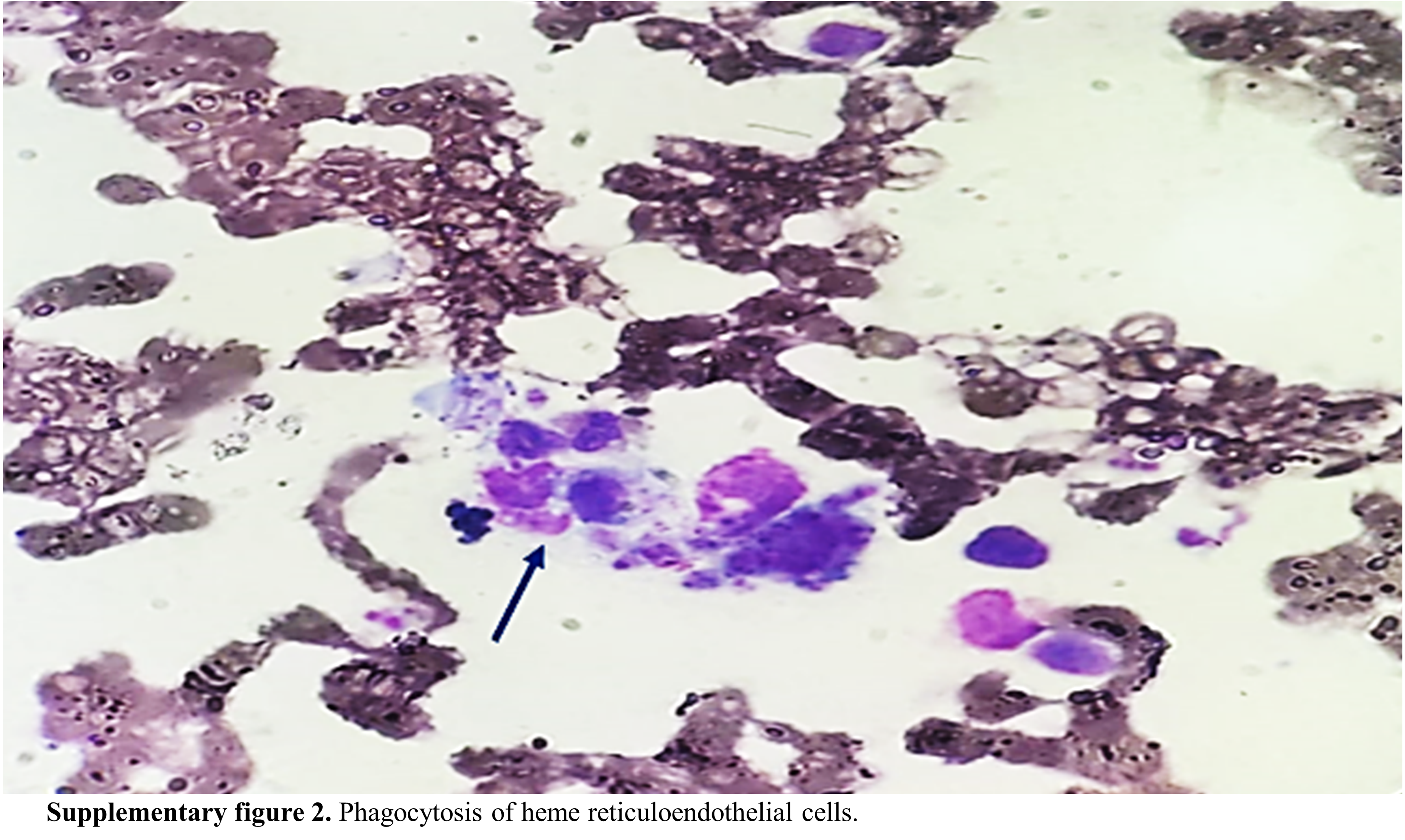

Supplement: Supplementary file 2 [file Image2.tif]
